# Structural Determinants for Substrate Binding and Catalysis in Triphosphate Tunnel Metalloenzymes*

**DOI:** 10.1074/jbc.M115.674473

**Published:** 2015-07-28

**Authors:** Jacobo Martinez, Vincent Truffault, Michael Hothorn

**Affiliations:** From the ‡ Structural Plant Biology Laboratory, Department of Botany and Plant Biology, University of Geneva, 1211 Geneva, Switzerland and; § Department of Biochemistry, Max Planck Institute for Developmental Biology, 72076 Tübingen, Germany

**Keywords:** adenylate cyclase (adenylyl cyclase), enzyme mechanism, manganese, nuclear magnetic resonance (NMR), protein structure, x-ray crystallography, inorganic polyphosphatase, thiamine triphosphatase

## Abstract

Triphosphate tunnel metalloenzymes (TTMs) are present in all kingdoms of life and catalyze diverse enzymatic reactions such as mRNA capping, the cyclization of adenosine triphosphate, the hydrolysis of thiamine triphosphate, and the synthesis and breakdown of inorganic polyphosphates. TTMs have an unusual tunnel domain fold that harbors substrate- and metal co-factor binding sites. It is presently poorly understood how TTMs specifically sense different triphosphate-containing substrates and how catalysis occurs in the tunnel center. Here we describe substrate-bound structures of inorganic polyphosphatases from *Arabidopsis* and *Escherichia coli*, which reveal an unorthodox yet conserved mode of triphosphate and metal co-factor binding. We identify two metal binding sites in these enzymes, with one co-factor involved in substrate coordination and the other in catalysis. Structural comparisons with a substrate- and product-bound mammalian thiamine triphosphatase and with previously reported structures of mRNA capping enzymes, adenylate cyclases, and polyphosphate polymerases suggest that directionality of substrate binding defines TTM catalytic activity. Our work provides insight into the evolution and functional diversification of an ancient enzyme family.

## Teaser

**Background:** Triphosphate tunnel metalloenzymes carry out diverse enzymatic reactions.

**Results:** Two metal co-factors are identified involved in substrate binding and in catalysis.

**Conclusion:** A unified catalytic mechanism is proposed and biochemically investigated.

**Significance:** The functional diversity of TTM enzymes is rationalized by a common mechanism that allows very different substrates to be bound and processed.

## Introduction

Inorganic polyphosphate (polyP) is a linear polymer of orthophosphate units joined by phosphoanhydride bonds. It occurs ubiquitously and abundantly in all life forms (1). In bacteria, polyP kinases generate polyP from ATP, but it is presently unknown how the cellular polyP stores of higher organisms are being synthesized (2, 3). We previously reported a fungal polyP polymerase that is distinct from bacterial polyP kinases (4, 5). The yeast enzyme resides in the vacuolar transporter chaperone (VTC)^2^ membrane protein complex, which generates polyP from ATP in the cytosol and translocates the growing chain into the vacuole (4, 6). The catalytic core of VTC maps to a cytoplasmic 8-stranded β-tunnel domain in Vtc4p, which harbors binding sites for the nucleotide substrate, for a manganese metal co-factor and for an orthophosphate priming the polymerase reaction (4).

The Vtc4p β-tunnel domain is not unique to eukaryotic polyP polymerases but is a structural hallmark of triphosphate tunnel metalloenzymes (TTMs), whose characteristic features are the presence of a topologically closed hydrophilic β-barrel, the preference for triphosphate-containing substrates, and for a divalent metal co-factor (PFAM (7) families CYTH (8) and VTC (9, 10)). Founding members of the TTM family were fungal (11, 12), protozoal (9), and viral RNA triphosphatases (13–16). Subsequently, other enzymes with very similar tunnel topologies were discovered, including the bacterial class IV adenylate cyclase cyaB (17, 18), mammalian thiamine triphosphatases (ThTPases) (19–21), and long- (22) and short-chain (23–26) inorganic polyphosphatases. Finally, the tunnel domain fold is also found in the mediator head complex subunits Med18 and Med20, but these proteins appear to have lost catalytic activity (27).

A staggering number of different catalytic activities and substrate preferences has been reported within the TTM family (10). However, the lack of substrate- and product-bound structures has made it difficult to define the sequence-fingerprints responsible for a specific enzyme activity in individual family members.

## Experimental Procedures

### 

#### 

##### Protein Expression and Purification

AtTTM3 (Uniprot ID Q9SIY3) was cloned into vector pMH-HT providing an N-terminal His_6_ tag and a tobacco etch virus protease cleavage site. Protein expression in *Escherichia coli* BL21 (DE3) RIL to *A*_600 nm_ = 0.6 was induced with 0.25 mm isopropyl β-d-galactoside in terrific broth at 16 °C for 16 h. Cells were collected by centrifugation at 4500 × *g* for 30 min, washed in PBS buffer, centrifuged again at 4500 × *g* for 15 min, and snap-frozen in liquid nitrogen. For protein purification cells were resuspended in lysis buffer (50 mm Tris-Cl (pH 8.0), 500 mm NaCl, 5 mm 2-mercaptoethanol), homogenized (Emulsiflex C-3, Avestin), and centrifuged at 7000 × *g* for 60 min. The supernatant was loaded onto a Ni^2+^ affinity column (HisTrap HP 5 ml, GE Healthcare), washed with 50 mm Tris (pH 8), 1 m NaCl, 5 mm 2-mercaptoethanol, and eluted in lysis buffer supplemented with 200 mm imidazole (pH 8.0). The His_6_ tag was cleaved with tobacco etch virus for 16 h at 4 °C during dialysis against lysis buffer. AtTTM3 was further purified by a second Ni^2+^ affinity step and by gel filtration on a Superdex 75 HR26/60 column (GE Healthcare) equilibrated in 25 mm Tris (pH 7.2), 250 mm NaCl, 5 mm 2-mercaptoethanol. Monomeric peak fractions were dialyzed against 20 mm Hepes (pH 7.4), 50 mm NaCl, 0.5 mm tris(2-carboxyethyl)phosphine and concentrated to 35 mg/ml for crystallization. Protein concentrations were estimated by protein absorption at 280 nm using the calculated molecular extinction coefficient. Site-specific mutations were introduced by PCR, and mutant proteins were purified like wild type.

The coding sequence of ygiF was amplified from genomic DNA (*E. coli* Mach 1 cells, Life Technologies) by PCR, and a synthetic gene coding for full-length mouse ThTPase (Uniprot ID Q8JZL3) and codon-optimized for expression in *E. coli* was obtained from Geneart (Life Technologies). Coding sequences were cloned into plasmid pMH-HT, and expression and purification were performed as described for AtTTM3.

##### Crystallization and Data Collection

Tetragonal AtTTM3 crystals (form A) developed at room temperature from hanging drops composed of 1.5 μl of protein solution and 1.5 μl of crystallization buffer (2.6 m NaCl, 0.1 m citric acid/NaOH (pH 5.0)) suspended over 1.0 ml of the latter as reservoir solution. Crystals were transferred in reservoir solution supplemented with 20% (v/v) ethylene glycol and 0.5 m KI for 20 s and snap-frozen in liquid nitrogen. Single-wavelength anomalous diffraction (SAD) data were collected on a Rigaku MicroMax rotating anode equipped with a copper filament, osmic mirrors, and an R-AXIS IV++ detector. Subsequently, an isomorphous native dataset was collected on a crystal originating from the same crystallization drop (see Table 1). A monoclinic crystal form (form B) developed in 20% (w/v) PEG 3350, 0.2 m NaCl, 0.1 m Bis-Tris (pH 7.0) and diffracted up to 1.3 Å resolution. Data processing and scaling were done with XDS (November 2014 version) (28). Hexagonal crystals for full-length ygiF grew at room temperature in hanging drops (1.5 μl and 1.5 μl) containing 0.2 m NaCl, 20 (w/v) PEG 3350. Crystals were transferred into crystallization buffer supplemented with 20% (v/v) ethylene glycol and snap-frozen in liquid nitrogen. For phasing, 0.1 m NaI was added to the cryo solution, and crystals were soaked for 5 min. SAD data were collected at a wavelength of 1.9 Å (see Table 2). Tetragonal mouse ThTPase crystals (form A) developed at room temperature from hanging drops composed of 1.5 μl of protein solution and 1.5 μl of crystallization buffer (27% (w/v) PEG 3350, 0.1 m Tris (pH 9.0), 0.2 m MgCl_2_) suspended over 1.0 ml of the latter as reservoir solution. Crystals were transferred into a reservoir solution supplemented with 20% (v/v) ethylene glycol and snap-frozen in liquid nitrogen. Monoclinic crystals (form B) developed in 1.6 m sodium/potassium phosphate buffer pH 6.8 (1:1 ratio) and were cryo-protected by stepwise transfer into a solution containing 1.6 m sodium/potassium phosphate buffer pH 6.8 and 20% (v/v) ethylene glycol.

##### Co-crystallization and Soaking Experiments

AtTTM3-PPP_i_-Mg^2+^/Mn^2+^ form A crystals were transferred into the soaking solution (2.6 m NaCl, 0.1 Bis-Tris (pH 5), 10 mm sodium tripolyphosphate (Sigma), 10 mm MgCl_2_ (or MnCl_2_), 20% (v/v) ethylene glycol) by serial transfer to replace the citrate otherwise bound to the tunnel center.

For AtTTM3-PPP_i_-Mg^2+^/Mn^2+^, form B crystals were soaked for 20–30 min in 20% (w/v) PEG 3350, 0.2 m NaCl, 0.1 m Bis-Tris pH 7.0, 20% (v/v) ethylene glycol, 10 mm sodium tripolyphosphate, 5 mm MnCl_2_ using the same procedure as outlined above (substrate-bound structure).

For AtTTM3-P_i_-Mn^2+^, AtTTM3 was co-crystallized with 5 mm sodium tripolyphosphate and 10 mm MnCl_2_ (product-bound structure).

For ygiF-PPP_i_-Mg^2+^/Mn^2+^, the substrate-bound complex was obtained by soaking (20–30 min) ygiF apo crystals in their crystallization buffer supplemented with 20% (v/v) glycerol, 10 mm sodium tripolyphosphate and 5 mm MgCl_2_ or MnCl_2_. ThTPase-ThTP-Mg^2+^. Form B crystals were soaked in crystallization buffer containing 20% (v/v) glycerol, 10 mm thiamine triphosphate and 10 mm MgCl_2_.

For ThTPase-ThDP-Mg^2+^, the product-bound structure was obtained by soaking form A crystals in a solution containing 10 mm sodium tripolyphosphate and 10 mm MnCl_2_ for 30 min. Two data sets were collected, one with λ = 1.0 Å and one close to the Mn-K edge (λ = 1.9 Å). No anomalous signal was found in the latter data set, possibly due to the high MgCl_2_ (0.2 m) concentration present in the crystallization buffer.

##### Structure Solution and Refinement

To solve the structure of AtTTM3, SAD and native data were scaled together with the program XPREP (Bruker AXS, Madison, WI) for SIRAS (single isomorphous replacement with anomalous scattering) phasing. The program SHELXD (29) was used to locate 37 iodine sites. Consistent sites were input in the program SHARP (30) for site refinement and phasing at 3.0 Å resolution. Density modification and phase extension to 2.6 Å was carried out with PHENIX.RESOLVE (31). The structure was built in alternating cycles of model correction in COOT (32) and restrained refinement in refmac5 (33) against a high resolution native data set (Table 1). The structure of crystal form B was determined by molecular replacement with the program PHASER (34).

**TABLE 1:** **Crystallographic data collection and refinement statistics for AtTTM3** Highest resolution shell is shown in parenthesis.

	AtTTM3 NaI soak (Co K_α_)	AtTTM3/PPP_i_/Mg^2+^ (form A)	AtTTM3/PPP_i_/Mn^2+^ (form A)	AtTTM3/PPP_i_/Mn^2+^ (form A; Mn-K edge)	AtTTM3/PPP_i_/Mn^2+^ (form B)	AtTTM3/PPP_i_/Mn^2+^ (form B, Mn-K edge)	AtTTM3/P_i_/Mn^2+^
PDB ID		5a5y	5a66		5a67		5a68

**Data collection**							
Beam line	In house	SLS PXII	SLS PXII	SLS PXII	SLS PXIII	SLS PXIII	SLS PXII
Wavelength (Å)	1.54	1.0	1.0	1.8	1.0	1.82	1.0
Space group	*I422*	*I422*	*I422*	*I422*	*P2_1_*	*P2_1_*	*P2_1_*
Cell dimensions							
*a, b*, *c* (Å)	136.03, 136.03, 145.64	136.33, 136.33, 144.20	136.27, 136.27, 145.48	136.32, 136.32, 145.99	44.37, 33.87, 72.22	44.36, 33.87, 72.32	44.22, 33.81, 71.81
α, β, γ (°)	90, 90, 90	90, 90, 90	90, 90, 90	90, 90, 90	90, 94.96, 90	90, 94.98, 90	90, 94.4, 90
Resolution (Å)	19.76-2.60	45.33-1.92	48.18-2.05	48.20-2.76	19.61-1.30	44.19-2.52	43.20-1.67
	(2.76-2.60)	(2.04-1.92)	(2.17-2.05)	(2.93-2.76)	(1.37-1.30)	(2.67-2.52)	(1.77-1.67)
*R*_sym_	0.145 (0.763)	0.076 (1.212)	0.088 (0.74)	0.266 (1.117)	0.046 (0.772)	0.038 (0.061)	0.085 (0.615)
*I*/σ*I*	12.11 (1.96)	20.06 (2.18)	19.13 (2.20)	12.02 (1.82)	20.04 (1.94)	25.29 (15.68)	9.5 (1.73)
Completeness (%)	98.8 (93.9)	98.5 (90.8)	98.0 (88.1)	98.1 (88.6)	98.3 (93.3)	98.4 (95.0)	94.4 (76.8)
Redundancy	5.02 (3.75)	10.59 (8.35)	11.73 (6.47)	8.34 (6.52)	6.24 (5.27)	3.30 (3.08)	4.1 (2.95)

**Refinement**							
Resolution (Å)		45.33-1.92	48.18–2.05		19.61-1.30		43.20-1.67
No. reflections		46,150	38,451		50,172		21,931
*R*_work_/*R*_free_		0.153/0.198	0.182/0.220		0.138/0.174		0.182/0.222
No. atoms							
Protein		3455	3455		1775		1670
P_i_/PP_i_/PPP_i_		26	26		13		10
Mg^2+^/Mn^2+^		2	2		2		3
Water		239	227		248		135
B-factors							
Protein		38.60	30.07		15.07		15.65
P_i_/PP_i_/PPP_i_		34.78	29.41		18.34		34.16
Mg^2+^/Mn^2+^		36.00	29.97		20.50		21.43
Water		44.82	40.89		35.33		39.39
r.m.s.d.							
Bond lengths (Å)		0.007	0.006		0.013		0.010
Bond angles (°)		1.268	1.098		1.377		1.486
Ramachandran plot (%)							
Favored		99.3	99.3		100		100
Outliers		0	0		0		0

The structure of ygiF was solved by scaling redundant SAD data using XPREP. SHELXD located 15 consistent iodine and sulfur sites, which were input into SHARP for SAD site refinement and phasing at 2.7 Å resolution. Density modification and phase extension to 2.15 Å was carried out with PHENIX.RESOLVE (Table 2). The structure of mouse ThTPase was solved using the molecular replacement methods as implemented in the program PHASER and using the structure of the human ThTPase (PDB ID 3BHD) as a search model. Analysis with MolProbity (35) indicated excellent stereochemistry for all refined models. Phasing and refinement statistics are summarized in Tables 1–3.

**TABLE 2:** **Crystallographic data collection and refinement statistics for ygiF** Highest resolution shell is shown in parenthesis.

	ygiF (NaI soak)	ygiF/PPP_i_/Mg^2+^	ygiF/PPP_i_/Mn^2+^	ygiF/PPP_i_/Mn^2+^ (Mn-K edge)
PDB ID		5a60	5a61	

**Data collection**				
Beam line	SLS PXII	SLS PXII	SLS PXII	SLS PXII
Wavelength (Å)	1.9	1.0	1.0	1.7
Space group	*P6*_5_	*P6*_5_	*P6*_5_	*P6_5_*
Cell dimensions				
a = b, c (Å)	91.11, 125.56	89.92, 125.58	89.68, 125.33	89.95, 125.31
α, β, γ (°)	90, 90, 120	90, 90, 120	90, 90, 120	90, 90, 120
Resolution (Å)	49.127-2.70	48.88-1.82	48.77-1.50	48.74-1.86
	(2.87-2.70)	(1.93-1.82)	(1.59-1.50)	(1.97-1.86)
*R*_sym_	0.077 (0.186)	0.068 (0.944)	0.036 (1.20)	0.037 (0.313)
*I*/σ*I*	31.08 (11.28)	14.91 (2.18)	23.90 (1.93)	26.80 (3.91)
Completeness (%)	95.8 (90.2)	98.8 (96.9)	99.7 (98.3)	97.9 (90.2)
Redundancy	19.63 (18.47)	9.70 (9.06)	15.48 (14.18)	8.30 (3.55)

**Refinement**				
Resolution (Å)		48.88-1.82	48.77–1.50	
No. reflections		47,520	86,573	
*R*_work/_ *R*_free_		0.150/0.189	0.151/0.171	
No. atoms				
Protein		3398	3395	
P_i_/PP_i_/PPP_i_		13	13	
Mg^2+^/Mn^2+^		2	2	
Water		289	295	
B-factors				
Protein		55.83	52.41	
P_i_/PP_i_/PPP_i_		46.83	42.84	
Mg^2+^/Mn^2+^		46.65	42.53	
Water		58.34	58.82	
r.m.s.d.				
Bond lengths (Å)		0.007	0.006	
Bond angles (°)		1.149	1.186	
Ramachandran plot (%)				
Favored		99.1	99.3	
Outliers		0	0	

**TABLE 3:** **Crystallographic data collection and refinement statistics for mouse ThTPase** Highest resolution shell is shown in parenthesis.

	mThTPase/ThTP/Mg^2+^	mThTPase/ThDP/P_i_/Mg^2+^
PDB ID	5a64	5a65

**Data collection**		
Beam line	ESRF ID29	SLS 5PXIII
Wavelength (Å)	0.98	1.0
Space group	*C2*	*P4_3_2_1_2*
Cell dimensions		
a, b, c (Å)	103.18, 93.58, 70.78	105.48, 105.48, 111.81
α, β, γ (°)	90, 93.04, 90	90, 90, 90
Resolution (Å)	70.68-2.10 (2.23-2.10)	49.40-1.98 (2.10-1.98)
*R*_sym_	0.059 (0.581)	0.11 (2.03)
*I*/σ*I*	19.34 (2.36)	19.66 (1.90)
Completeness (%)	99.5 (98.3)	99.7 (98.6)
Redundancy	4.58 (4.49)	25.60 (23.85)

**Refinement**		
Resolution (Å)	70.68-2.10	49.40-1.98
No. reflections	37,148	42,091
*R*_work/_ *R*_free_	0.212/0.232	0.208/0.258
No. atoms		
Protein	3102	3117
ThTP/P_i_	60	62
Mg^2+^/Mn^2+^	2	4
Water	148	142
B-factors		
Protein	13.54	38.56
P_i_/PP_i_/PPP_i_	50.08	60.08
Mg^2+^/Mn^2+^	44.18	43.38
Water	44.88	53.05
r.m.s.d.		
Bond lengths (Å)	0.012	0.010
Bond angles (°)	1.46	1.515
Ramachandran plot (%)		
Favored	98.0	99.0
Outliers	0	0

##### Synthesis of Thiamine Triphosphate

Thiamine triphosphate was synthesized as described (36) and purified by preparative high performance liquid chromatography.

##### NMR Time-course Experiment

A series of one-dimensional ^31^P NMR experiments was acquired at 310 K with a 600-MHz Bruker Avance-III spectrometer using a QXI probe-head allowing direct detection of ^31^P and equipped with a z-spoil gradient coil. ^31^P spectra were recorded using a relaxation delay of 1 s and an acquisition time of 42.6 ms (spectral width = 12,019.23 Hz). 128 scans were collected, resulting in a measurement time of 140 s per spectrum. 512 spectra were collected over a total acquisition time of ∼20 h. The enzymatic reaction was performed using 50 nm AtTTM3 and 5 mm sodium tripolyphosphate in 20 mm Bis-Tris propane (pH 8.5), 250 mm NaCl, 5 mm MgCl_2_ mixture. Deuterated water was added to a final concentration of 20% to the reaction mix before starting the experiment. Spectral parameters were calibrated and optimized on a 5 mm sodium tripolyphosphate sample to minimize time loss between the beginning of the reaction and the beginning of its observation by NMR. Spectra were processed using Topspin (version 2.1.6) (Bruker).

##### Phosphohydrolase Activities of AtTTM3 and ygiF Mutant Proteins

For the determination of the phosphohydrolase activity 2.5 nm AtTTM3 were incubated with 0.5 μm concentrations of the different phosphate-containing substrates in reaction buffer (150 mm NaCl, 20 mm Bis-Tris propane (pH 8.5), 5 mm MgCl_2_) at 37 °C. The reaction was stopped after 10 min, and the amount of free inorganic phosphate released was measured using the malachite green assay with minor modifications (37). 100 μl of reaction solution were mixed with 28 μl of dye mix (3 mm malachite green, 15% (v/v) sulfuric acid, 1.5% molybdate (w/v), 0.2% (v/v) Tween 20). After 5 min of incubation with the dye, the absorption at 595 nm was measured using a synergy H4 plate reader (Biotek). For each substrate a blank reaction was prepared lacking the enzyme. Controls either contained EDTA to a final concentration of 5 mm, or the enzyme was heat-inactivated at 95 °C for 5 min. To compare wild-type and mutant versions of AtTTM3 and ygiF, enzyme concentrations were increased to 180 nm to detect residual activity for some of the mutant proteins. Experiments were performed in triplicate; average values are plotted ±S.D.

Thermal shift assays were performed as previously described (38). Sypro Orange (Sigma) was added to wild-type and mutant AtTTM3 proteins in 25 mm Tris (pH 8.0), 250 mm NaCl, 5 mm 2-mercaptoethanol mixed with Sypro Orange to a final protein concentration of 10 μm. The protein-dye mixtures were loaded into a 96-well RT-PCR plate (Thermo Scientific), and measurements were performed using a C1000 thermal cycler (Bio-Rad). The fluorescence of SYPRO Orange was continously monitored at 570 nm, as a temperature gradient was applied (0.05 °C/s from 10 °C to 95 °C). Data were analyzed using the CFX Manager software (Bio-Rad). The maximum of the first derivative for each melting curve indicates the melting point of the protein. Experiments were performed in triplicate; average values are plotted ±S.D.

##### Polyphosphate Detection by UREA PAGE

Reactions contained 50 μm concentrations of the respective enzyme (AtTTM3, ygiF, mouse ThTPase, Vtc4p) in 150 mm NaCl, 20 mm Bis-Tris propane (pH 8.3), 1 mm MnCl_2_, and 10 mm ATP as substrate. Reactions were incubated at room temperature overnight. The reaction was stopped by adding Proteinase K (Sigma), and the resulting samples were mixed 1:1 with sample buffer (50% (w/v) urea, 2× Tris borate-EDTA, 20 mm EDTA (pH 8.0), 20% (v/v) glycerol, 0.25% (w/v) bromphenol blue). Samples were loaded onto a Tris borate-EDTA (TBE)-urea polyacrylamide gel (1× TBE, 7 m urea, 15% (v/v) polyacrylamide (19:1 acrylamide/bisacrylamide), 0.06% (w/v) tetramethylethylenediamine, 0.6% (w/v) ammonium persulfate) and stained with 4′,6-diamidino-2-phenylindole dihydrochloride (DAPI).

## Results and Discussion

**FIGURE 1.**
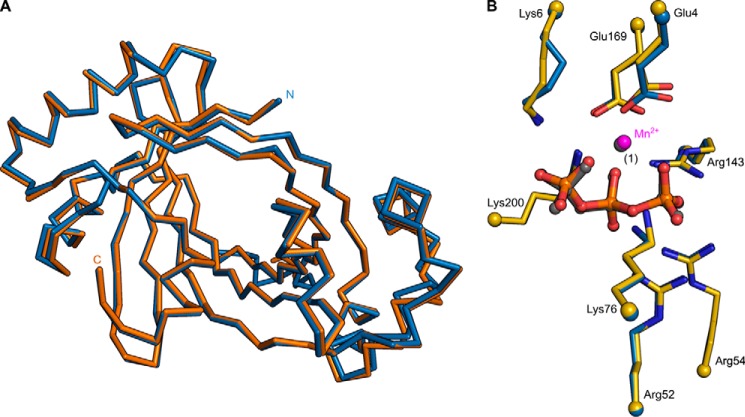
*A*, structural superposition of AtTTM3 crystals forms A (*blue*) and B (*orange*). The structures align with an r.m.s.d. of 0.5 Å comparing 202 corresponding C_α_ atoms. *B*, close-up of the tunnel domain centers in forms A and B (with selected side chains shown in bond representation), both bound to PPP_i_ (in bond representation) and Mn^2+^ (*magenta*/*gray spheres*).

**FIGURE 2.**
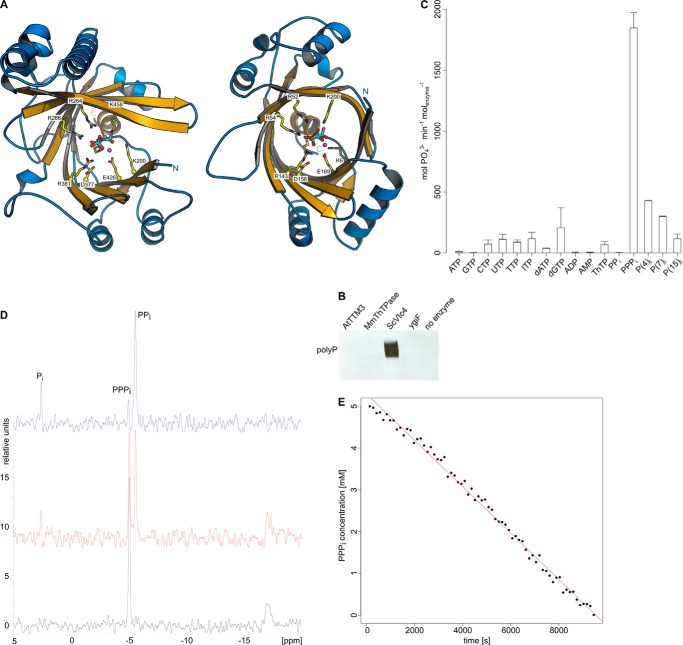
**AtTTM3 is a short-chain inorganic polyphosphatase.** *A*, ribbon diagrams of the yeast inorganic polyphosphate polymerase Vtc4p (*left panel*) and *Arabidopsis* TTM3 (*right panel*). Tunnel domain β-strands are shown in *yellow*, and the α-helices are in *blue*. The TTM domain is topologically closed on one side by a C-terminal plug helix. Conserved residues in the tunnel center are shown (in bond representation, in *yellow*) alongside with the triphosphate substrates and Mn^2+^ ions (*magenta spheres*). *B*, DAPI stained UREA page gel reveals that only Vtc4p can generate inorganic polyphosphate from ATP. *C*, substrate specificity of AtTTM3. P_i_ release is measured for a range of different di- and triphosphate containing potential substrates. *D*, a one-dimensional ^31^P NMR time-course experiment reveals that AtTTM3 specifically generates PP_i_ and P_i_ from PPP_i_. *t* = 0 min (*black line*), 94 min (*red line*), and 141 min (*blue line*). *E*, decay of the PPP_i_ substrate concentration during the NMR time-course experiment indicates a turnover number of ∼10/s.

Our initial aim was to identify a polyP polymerase in plants using a combined structural and biochemical screen. We located three putative TTM proteins in *Arabidopsis thaliana*. AtTTM3 shares 12% sequence identity with Vtc4p (r.m.s.d. is 2.6 Å, comparing 149 corresponding C_α_ atoms and 1.1 Å comparing 77 C_α_ atoms in the tunnel center) and contains a conserved β-tunnel domain (26). We determined co-crystal structures of AtTTM3 with tripolyphosphate and Mg^2+^ or Mn^2+^ metal co-factors in two independent crystal lattices (Fig. 1). Crystal form B corresponds to the previously reported structure of AtTTM3 (PDB ID 3v85) in complex with a citric acid molecule (r.m.s.d is 0.3 Å comparing 203 corresponding C_α_ atoms) (26). Our structures reveal a conserved mode of substrate and metal co-factor binding in AtTTM3 and Vtc4p and an invariant arrangement of residues in the tunnel center (Fig. 2*A*) (4). We thus tested whether AtTTM3 can polymerize polyP from ATP or other nucleotide substrates but could not detect such an activity (Fig. 2*B*). Instead, AtTTM3 has specific, Mg^2+^ ion-dependent, short-chain polyphosphatase activity (Fig. 2*C*) as previously reported (26). We find that AtTTM3 hydrolyzes tripolyphosphate (PPP_i_) into pyro (PP_i_)- and orthophosphate (P_i_) with a turnover rate of ∼10/s (Fig. 2, *D* and *E*). The P_i_ release from PPP_i_ amounts to 27 ± 6/s when assayed by malachite green (see “Experimental Procedures”). AtTTM3 is not able to hydrolyze PP_i_ but appears to catalyze the asymmetric cleavage of inorganic polyP*_n_* (*n* = 3–15), releasing P_i_ (Fig. 2, *C* and *D*).

### 

#### 

##### Plant and Bacterial Tripolyphosphatases Contain Two Metal Coordination Sites

**FIGURE 3.**
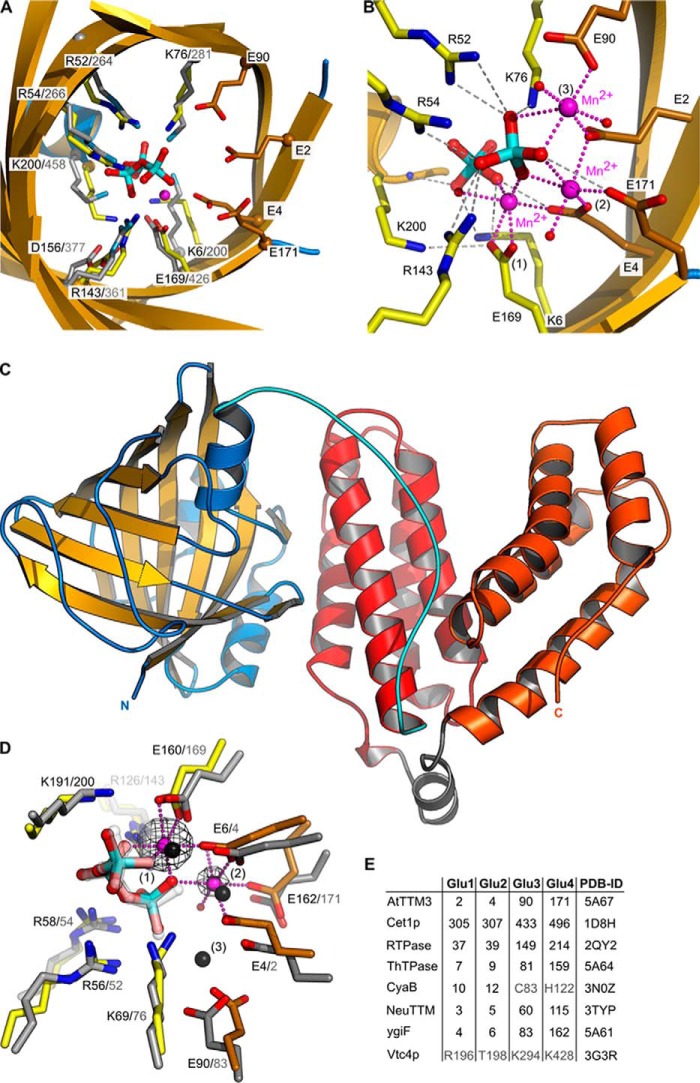
**An acidic patch in TTM proteins allows for the coordination of two metal ions.** *A*, structural superposition of AtTTM3 (in *yellow*) and Vtc4p (in *gray*) (r.m.s.d. is 1.1 Å comparing 77 corresponding C_α_ atoms in the tunnel center) identifies an acidic patch in AtTTM3 (in *orange*, in bond representation), which is absent Vtc4p. The AtTTM3 tripolyphosphate substrate and a Mn^2+^ ion (*magenta sphere*) are shown alongside. *B*, structure of a AtTTM3 post-catalysis complex reveals two orthophosphates and three Mn^2+^ ions bound to the tunnel center. *C*, ribbon diagram of full-length ygiF from *E. coli*. The tunnel domain (residues 1–200) is shown in *yellow* (β-strands) and *blue* (α-helices), the linker region (residues 201–220) is highlighted in *cyan*, and the four-helix bundles of the C-terminal CHAD domain are depicted in *red* (residues 221–324) and *orange* (residues 383–433), respectively. *D*, structural superposition (r.m.s.d. is 0.65 Å comparing 75 corresponding C_α_ atoms) of the ygiF tunnel core (in *yellow*, the acidic patch is in *orange*) bound to PPP_i_ and Mn^2+^ ions (*magenta spheres*) with the AtTTM3 product complex (in *gray*). A phased omit difference density map contoured at 25 σ is shown alongside (*black mesh*). Note that two of the three AtTTM3 metal coordination sites are also found in ygiF. *E*, table comparison of acidic patch residues in different TTM proteins. *RTPase*, RNA triphosphatase.

**FIGURE 4.**
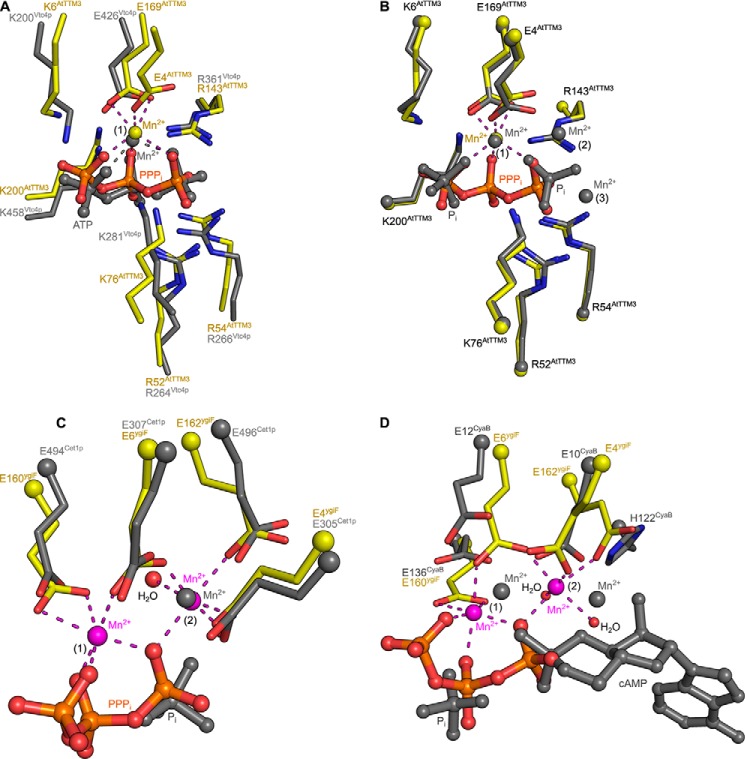
**TTM proteins with different enzymatic activities harbor two metal ion centers.** *A*, structural superposition of a AtTTM3-PPP_i_-Mn^2+^ complex (in *yellow*, in bond representation) with the Vtc4p-ANP-Mn^2+^ complex (PDB ID 3G3R, in *gray*) (r.m.s.d. is 2.7 Å comparing 151 corresponding C_α_ atoms) reveals a similar mode of substrate and metal co-factor binding to site 1. Glu-4 from the acidic patch in AtTTM3 is, however, not found in Vtc4p. *B*, comparison of AtTTM3-PPP_i_-Mn^2+^ (in *yellow*) with the product bound state (r.m.s.d. is 0.3 Å comparing 203 corresponding C_α_ atoms) reveals that the two orthophosphates in the product complex align with the terminal phosphates of the PPP_i_ substrate. One of the three Mn^2+^ ions (bound to site 1) is found consistently in both structures. *C*, structural superposition (r.m.s.d. is 2.2 Å comparing 138 corresponding C_α_ atoms) of ygiF bound to PPP_i_ and to 2 Mn^2+^ ions located in sites 1 and 2 with the RNA triphosphatase Cet1p (PDB ID 1D8H, in *gray*) reveals that the Cet1p manganese ion previously reported maps to metal binding site 2. *D*, comparisons of the ygiF-PPP_i_-Mn^2+^ complex with the product-bound state of the adenylate cyclase cyaB (PDB ID 3N10, in *gray*) again reveals two conserved metal binding sites. The Mn^2+^ ion bound to site 2 in the case of cyaB is coordinated by His-122.

Comparison of AtTTM3 and Vtc4p revealed that four basic residues, which lead the growing polyP chain away from the active site in Vtc4p, are specifically replaced by four glutamate residues (Glu-2^AtTTM3^, Glu-4^AtTTM3^, Glu-90^AtTTM3^, and Glu171^AtTTM3^) in AtTTM3 (Fig. 3, *A* and *E*) (4). These residues, which form an acidic patch in the vicinity of the AtTTM3 tripolyphosphate substrate, are conserved among diverse TTM proteins with different catalytic activities (Figs. 3*E* and 4). The corresponding residues in the RNA triphosphatase Cet1p (Glu305^Cet1p^, Glu307^Cet1p^, and Glu-496^Cet1p^) coordinate a Mn^2+^ ion (11), and mutation of these residues to alanine or aspartate impairs catalysis in fungal (12, 39), viral (13–16, 40,), and protozoan (9, 41) RNA triphosphatases, in a broad-range polyphosphatase from *Clostridium thermocellum* (22) and in a TTM adenylate cyclase (18).

Based on kinetic and mutational studies, one (23) and two-metal (40, 41) mechanisms have been proposed for TTMs, but only a few substrate complexes in the presence of metal co-factors have been reported thus far (4, 18). As AtTTM3 efficiently hydrolyzes PPP_i_ in the presence of Mg^2+^ ions only at neutral or basic pH, we used two crystal forms grown at pH 5.0 (form A) and pH 7.5 (form B) to crystallize substrate-metal complexes under non-hydrolyzable and hydrolyzable conditions, respectively. In both crystal forms, we determined structures of AtTTM3 in the presence of PPP_i_ and either MgCl_2_ or MnCl_2_. In all our structures the catalytic Mg^2+^ ions can be substituted by Mn^2+^ ions, for which we calculated phased anomalous difference electron density maps from data collected near the Mn-K edge to confirm their modeled positions (Tables 1 and 2). At pH 5.0 (form A) we find a triphosphate moiety bound in the AtTTM3 active site. Here, PPP_i_ coordinates a Mg^2+^/Mn^2+^ ion that acts as a bridge between the substrate and the invariant Glu-169^AtTTM3^ (Fig. 3*A*). The same metal co-factor coordination has been previously found in a Vtc4p AppNHp-Mn^2+^ complex (4) (Figs. 3*A* and 4*A*). A short soak at pH 7.0 (form B) shows the same arrangement of PPP_i_ and Mn^2+^ in the active site (Fig. 1). However, when we co-crystallized AtTTM3 with its substrate at neutral pH, we found PPP_i_ hydrolyzed and the tunnel center occupied by two orthophosphates and three Mn^2+^ ions (Fig. 3*B*). One of these Mn^2+^ ions is again found coordinated by the two phosphates (which correspond to the α and γ positions in PPP_i_; Fig. 4*B*) and by Glu-169^AtTTM3^, whereas the remaining metal coordination centers are formed by Glu-2^AtTTM3^, Glu-4^AtTTM3^, Glu-90^AtTTM3^, and Glu171^AtTTM3^ from the acidic patch conserved among many TTMs (Fig. 3, *B* and *E*).

To assess the functional relevance of the observed metal co-factors in AtTTM3, we studied substrate binding and metal ion coordination in the evolutionary distant TTM ygiF from *E. coli*. YgiF shares ∼18% sequence identity with AtTTM3 and has the same catalytic activity and substrate specificity (25, 26). The structure of full-length ygiF was solved using iodide/sulfur SAD phasing and reveals the conserved TTM fold (r.m.s.d. with the AtTTM3 tunnel domain is 2.0 Å compared with 172 corresponding C_α_ atoms, and 0.65 Å comparing 75 corresponding C_α_ in the tunnel center) (Fig. 3*C*). ygiF contains a second, α-helical domain, which is connected to the TTM by a well ordered linker (residues 201–221, *cyan* in Fig. 3*C*) and which has been previously annotated as CHAD domain (8). The domain folds into two four-helix bundles of similar architecture and connectivity forming a V-shaped assembly (shown in *red* and *orange* in Fig. 3*C*). A structural homology search with the program DALI (42) returned no significant hits, and the molecular function of this domain, which is often found in bacterial metallophosphoesterases (8), remains to be elucidated.

We next solved structures of ygiF in complex with PPP_i_ and in the presence of either MgCl_2_ or MnCl_2_ (Table 2). The ygiF triphosphate substrate is bound in the same conformation as observed in AtTTM3, and we could identify two metal co-factors. One Mg^2+^/Mn^2+^ ion is again found coordinated by tripolyphosphate and by Glu-160^ygiF^, which corresponds to Glu-169 in AtTTM3 (*position 1* in Fig. 3*D*). The second Mg^2+^/Mn^2+^ ion is coordinated by three glutamate residues from the acidic patch in ygiF (Glu-4^ygiF^, Glu-6^ygiF^, Glu-162^ygiF^) and by a water molecule that is positioned close to a terminal phosphate of the tripolyphosphate substrate (*position 2*, Fig. 3, *D* and *E*). When we calculated phased anomalous difference maps from diffraction data collected near the Mn-K edge, we found that the ygiF metal binding site 1 shows a higher occupancy compared with the second site (peak heights are 125 and 45 σ, respectively), whereas we cannot detect difference density at the third metal position found in AtTTM3 (Fig. 3*D*). Taken together, our experiments define two consistent Mg^2+^/Mn^2+^ coordination centers in plant and bacterial TTM tripolyphosphatases.

We compared our structures to other TTM enzymes for which metal ion-bound complexes have been reported. In the RNA triphosphatase Cet1p, a Mn^2+^ ion is bound by the acidic patch, and its position corresponds to site 2 in ygiF and AtTTM3 (Figs. 4*C* and 3*E*) (11). In a bacterial TTM protein with adenylate cyclase activity, both positions 1 and 2 are occupied by Mn^2+^ ions (Figs. 4*D* and 3*E*) (18). The acidic patch in mouse ThTPase (corresponding to site 2 in AtTTM3 and ygiF) again allows for Mg^2+^ ion binding, as concluded from NMR titration experiments (20). These findings together indicate that many TTM proteins contain two metal ion binding sites, as previously proposed (40, 41). It is likely that site 1 is generated by a triphosphate substrate-divalent metal ion complex binding to the tunnel center (21, 23). Here, the octahedral coordination of the Mg^2+^/Mn^2+^ ion is completed by an glutamate residue (Glu-169^AtTTM3^, Glu-160^ygiF^), which is invariant in all TTM proteins characterized thus far (Figs. 3*D* and 4). A second binding site is formed by three glutamate residues originating from an acidic patch conserved among many but not all TTM enzymes (Figs. 3, *A*, *D*, and *E*, and 4) (4, 12, 24). This metal ion is positioned close to a terminal phosphate in our AtTTM3 and ygiF substrate complex structures (Fig. 3*D*) and may thus be involved in catalysis rather than in substrate binding.

##### Structures of Mammalian Thiamine Triphosphatase Reveal the Location of the γ-Phosphate

**FIGURE 5.**
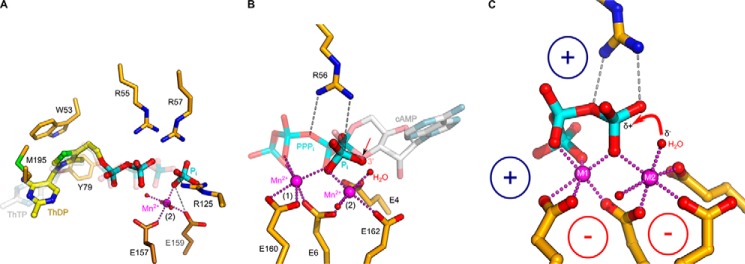
**TTM proteins use a two-metal catalytic mechanism.** *A*, close-up view of the mouse ThTPase tunnel center with either the ThTP substrate bound (transparent gray) or the ThDP/P_i_ products post-catalysis (in *yellow*, in bond representation). The thiamine part of ThTP is buried in a pocket close to the C-terminal plug helix of the TTM domain formed by Tyr-79 and Met-195; the thiazole ring makes a stacking interaction with Trp-53 (in *yellow*, in bond representation). The product P_i_ is coordinated by a Mn^2+^ ion bound to site 2 and by Arg-125. *B*, close-up of the ygiF active site (in *yellow*, in bond representation) bound to PPP_i_ and two Mn^2+^ ions (*magenta spheres*) in sites 1 and 2. The Mn^2+^ ion in site 2 coordinates a water molecule (*red sphere*), which is well positioned to act as nucleophile. Structural superposition with a product-bound class IV adenylate cyclase (PDB ID 3N10) reveals the O3′ of cAMP in the same position as the water molecule in ygiF. This position is also occupied by an oxygen atom of the product P_i_ located in the AtTTM3 post-catalysis complex. *C*, the suggested mechanism for acidic-patch containing TTM proteins. The metal ion in site 1 coordinates the triphosphate moiety of the substrate to the tunnel center by interacting with a conserved Glu residue. Three additional glutamates form metal binding site 2, which coordinates and polarizes a water molecule to attack the γ-phosphate of the substrate. Conserved basic residues in the tunnel center are involved in substrate binding and potentially stabilize the pyrophosphate leaving group.

We investigated the contributions of the two metal ion centers to TTM substrate binding and catalysis. A conceptual problem with the analysis of our plant and bacterial tripolyphosphatases is that they catalyze the asymmetric cleavage of a symmetric substrate (Fig. 2) (25, 26). It is thus difficult to assess in crystal structures, which terminal phosphate represents the γ-phosphate that is being hydrolyzed (Fig. 3, *A* and *B*). We thus structurally characterized a mammalian TTM ThTPase, which was previously shown to specifically hydrolyze thiamine triphosphate (ThTP) into ThDP and P_i_ (19–21, 43). We synthesized ThTP from ThDP and produced co-crystal structures of mouse ThTPase with its substrate at pH 6, where ThTPase catalytic activity is minimal (20). Consequently, we found an intact ThTP molecule bound in the tunnel center of ThTPase (Fig. 5*A*). The thiamine portion of the substrate binds to a pocket generated by the tunnel walls and the C-terminal plug helix, with the thiazole ring making a stacking interaction with Trp-53 and with Met-195 from the plug helix (Fig. 5*A*). The ThTP triphosphate moiety binds in the same conformation as outlined for the PPP_i_-bound structures of AtTTM3 and ygiF above. Our substrate-bound mouse ThTPase structure supports an earlier docking model of human ThTPase (21). We next solved a crystal structure of mouse ThTPase in the presence of ThTP and Mg^2+^ in a second crystal form grown at pH 9.0, where substrate hydrolysis can occur (20). Indeed, we found a product complex trapped in the active site of the enzyme, with a ThDP molecule and an orthophosphate located in the tunnel center (Fig. 5*A*). ThDP is coordinated by Arg-55 and Arg-57 in the substrate binding site but no longer allows for the coordination of a Mg^2+^/Mn^2+^ ion in metal binding site 1, possibly because the missing γ-phosphate would be required for Mg^2+^/Mn^2+^ coordination (Fig. 5*A*). The γ-phosphate in our structure apparently has been hydrolyzed, and the resulting P_i_ has slightly moved away from the tunnel center (Fig. 5*A*). It is now found coordinated by Arg-125 and in direct contact with a Mn^2+^ ion located in metal binding site 2, reinforcing the notion that this metal ion may play a crucial role in catalysis (Fig. 5*A*).

##### TTM Proteins Use a Two-metal Catalytic Mechanism

The structural features surrounding metal binding site 2 in our structures allow proposing a unified catalytic mechanism for triphosphate tunnel metalloenzymes. In our PPP_i_-bound ygiF structure we find a water molecule coordinated by the second Mn^2+^ ion, which is in an ideal position to serve as the activated nucleophile (Fig. 5, *B* and *C*). Indeed, structural superposition with a bacterial adenylate cyclase (18) reveals that its cAMP O3′ group, which acts as the nucleophile in the cyclization reaction, is located in the same position as the water molecule in our ygiF structure (Fig. 5*B*). Consistently, this position also is occupied by an oxygen atom of a product orthophosphate, which we located in our AtTTM3 post-catalysis structure (see above, Figs. 3*B* and 5*B*). Based on these findings, we propose that metal binding site 1 is required for proper substrate coordination in TTM proteins (21, 23), and metal ion 2 activates a water molecule to allow for a nucleophilic attack on the triphosphate substrate (Fig. 5*C*). This would rationalize why the glutamate residues from the acidic patch, which are involved in the coordination of the second metal ion, are well conserved among TTM proteins (Figs. 3*E* and 4). The basic residues in the tunnel center appear to be mainly involved in substrate coordination (Figs. 2*A* and 3*A*); however, the invariant Arg-56^ygiF^ (Arg-52^AtTTM3^) possibly activates the substrate for the nucleophilic attack by forming a hydrogen bond with the oxygen atom connecting the β- and γ-phosphate of the substrate (Fig. 5, *B* and *C*). The suggested TTM reaction mechanism is reminiscent of the one found in mammalian type V adenylate cyclases (44) and in nucleic acid polymerases (45, 46) as previously speculated (8).

**FIGURE 6.**
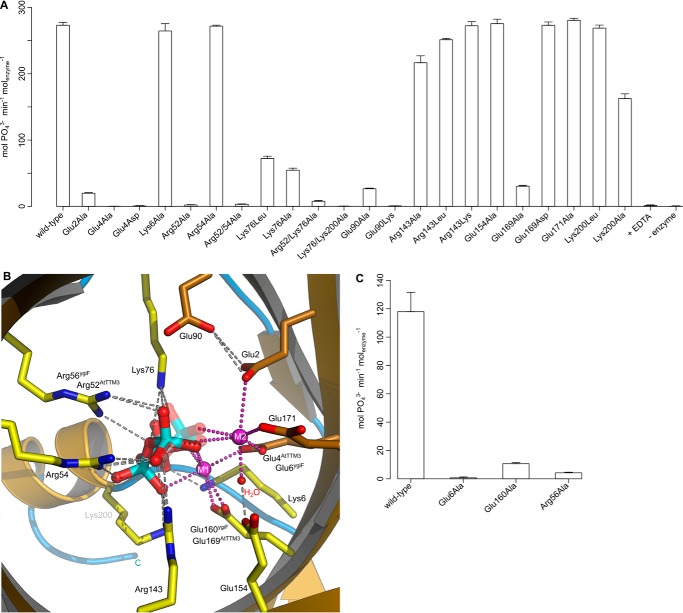
**Reduced enzyme activities for several AtTTM3 and ygiF mutant proteins are consistent with the proposed catalytic mechanism.** *A*, PPPase activity of structure-based point AtTTM3 point mutants found in direct contact with the PPP_i_ substrate or the two metal co-factors as shown in *B. C*, PPPase activity of the corresponding residues in ygiF.

**FIGURE 7.**
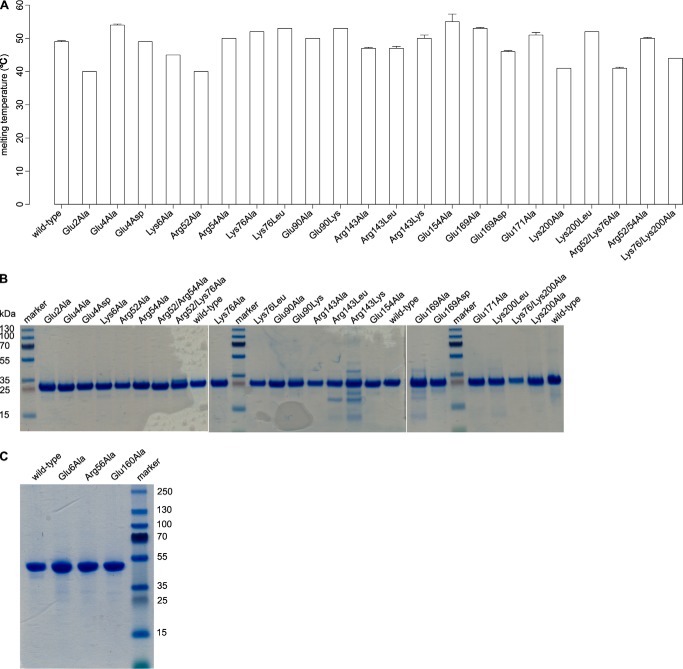
**Structural integrity and purity of AtTTM3 and ygiF mutant proteins.** *A*, melting temperatures for wild-type and mutant AtTTM3 recombinant proteins. SDS-PAGE analysis of purified wild-type and mutant AtTTM3 (*B*) and ygiF (*C*) proteins is shown. The calculated molecular weights for AtTTM3 and ygiF are 24.3 and 48.6 kDa, respectively.

We next performed a mutational analysis of substrate- and metal co-factor-interacting residues in AtTTM3 and ygiF (Fig. 6). Mutation of Glu-2^AtTTM3^, Glu-4^AtTTM3^, and Glu-169^AtTTM3^ to Asp or Ala strongly reduces the PPP_i_ hydrolysis of the plant enzyme (Fig. 6, *A* and *B*; see Fig. 7 for mutant protein stability). Consistently, changing the corresponding Glu-6^ygiF^ and Glu-160^ygiF^ to Ala impairs the enzymatic activity of ygiF, suggesting that the proper arrangement of metal binding sites 1 and 2 in the tunnel center is essential for catalysis (Fig. 6, *A–C*). Mutation of Arg-52^AtTTM3^ or Arg-56^ygiF^, but not of the neighboring Arg-54^AtTTM3^, to Ala again strongly inhibits catalysis, highlighting its potential role as proton donor (Fig. 6, *A–C*, see above). In addition, we find moderately reduced enzymatic activities upon the mutation of Lys-76^AtTTM3^ to Leu or Ala (Fig. 6, *A* and *B*). This residue and the corresponding Lys-69^ygiF^ appear to be involved in substrate binding and orient Glu-2^AtTTM3^/Glu-4^ygiF^ to coordinate metal ion 2 (Fig. 6, *A* and *B*). Glu-2^AtTTM3^ is also in contact with Glu-90^AtTTM3^, mutation of which to Ala again reduces the enzymatic activity of the *Arabidopsis* enzyme (Fig. 6, *A* and *B*). Taken together, our and previous mutational analyses (22, 24, 26) consistently suggest that the proper establishment of two metal centers appears to be critical for catalysis in TTM tripolyphosphatases and other TTM proteins (40, 41). The basic residues, with the exception of the catalytic Arg-52^AtTTM3^ appear to be mainly involved in substrate coordination in the tunnel center (Fig. 6, *A–C*). Further experimentation will be required to rationalize the specific effects of certain point mutations on substrate/metal co-factor binding or catalysis itself.

Comparison with Vtc4p suggests that the first but not the second metal binding site are present in the yeast polyphosphate polymerase (Figs. 2, *A* and *B*, and 4*A*) (4). Importantly, mutation of Lys-200^AtTTM3^, which corresponds to the catalytic Lys-458^Vtc4p^, to either Leu or Ala has little effect on tripolyphosphatase activity of the plant enzyme (Fig. 6, *A* and *B*) (4). This suggests that there are significant mechanistic differences between TTM polyphoshatases and polyphosphate polymerases despite their strong structural homology (Figs. 2, *A* and *B*, and 3*A*).

##### Directionality of Substrate Binding Defines TTM Catalytic Activity

**FIGURE 8.**
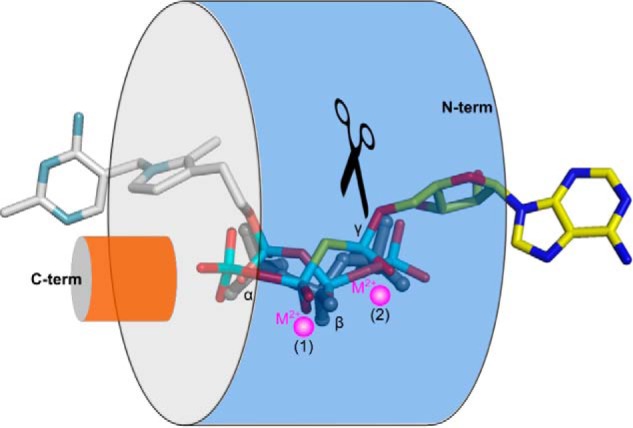
**Directionality of substrate binding defines TTM catalytic activity.** Schematic representation of PPP_i_ (ygiF), ThTP (mThTPase, in *gray*), and ATP-analog (PDB ID 3N0Y, in *yellow*) binding to the tunnel domain. Different substrates can bind to the tunnel from opposite sites. The respective triphosphate moieties are well aligned, and the cleavage site is maintained (*black scissors*), leading to different reaction products.

To better understand how acidic-patch containing TTMs are able to carry out very different enzyme reactions, we superimposed our substrate-bound structures of AtTTM3, ygiF, and mouse ThTPase with an ATP-analog complex of a bacterial TTM adenylate cyclase (18) (Fig. 8). We found that although the triphosphate parts of all ligands closely align in the tunnel center, their “tail” moieties can enter the tunnel domain from opposite sites in different enzymes (Fig. 8). The unique modes of substrate binding in ThTPases and adenylate cyclases allow these enzymes to carry out rather different reactions and to produce different leaving groups (ortho- and pyrophosphate, respectively) while maintaining a unified cleavage site in close proximity of metal binding site 2. Both the N- and C-terminal sides of the tunnel domain have evolved to recognize specific substrates, as exemplified by our ThTPase structure and by the class IV adenylate cyclase complexes (Figs. 5, *A* and *B*, and 8) (18). The observed substrate binding mode in mouse ThTPase reinforces the notion that to bind their substrates, TTMs require opening of their closed tunnel domains into a cup-shaped hand, as previously shown by NMR spectroscopy (20).

Members of the ancient triphosphate tunnel metalloenzyme family can be found in all kingdoms of life. Our comparative analysis defines that most of these enzymes share a common catalytic mechanism in their tunnel centers, yet they have evolved different substrate recognition modes on their tunnel entries. The observed substrate plasticity apparently allows TTM proteins to act on a wide array of enzyme substrates and to perform very different reactions, many of which likely remain to be discovered.

## Author Contributions

J. M. and M. H. designed the study and wrote the paper. V. T. performed NMR titrations, J. M. performed all other experiments. All authors analyzed data and approved the final version of the manuscript.

## Footnotes

2 The abbreviations used are:

VTC: vacuolar transporter chaperone
TTM: triphosphate tunnel metalloenzymes
AtTTM3: *A. thaliana* triphosphate tunnel metalloenzyme
PPP_i_: tripolyphosphate
ThTP: thiamine triphosphate
ThDP: thiamine diphosphate
ThTPase: thiamine triphosphatase
mThTPase: mouse ThTPase
SAD: single-wavelength anomalous diffraction
Bis-Tris: 2-[bis(2-hydroxyethyl)amino]-2-(hydroxymethyl)propane-1,3-diol
Bis-Tris propane: 1,3-bis[tris(hydroxymethyl)methylamino]propane
r.m.s.d.: root mean square deviation
Mn-K: manganese K x-ray absorption edge.
